# Role of Iron Availability in Modulating *Pseudomonas aeruginosa*’s Antifungal Effects on Planktonic and Biofilm Growth of *Scedosporium*/*Lomentospora* Under Cystic Fibrosis-Mimicking Conditions

**DOI:** 10.3390/jof12020089

**Published:** 2026-01-28

**Authors:** Thaís P. Mello, Iuri C. Barcellos, Simone S.C. Oliveira, Lucas Giovanini, Michaela Lackner, Marta H. Branquinha, André L.S. Santos

**Affiliations:** 1 Laboratório de Estudos Avançados de Microrganismos Emergentes e Resistentes (LEAMER), Departamento de Microbiologia Geral, Instituto de Microbiologia Paulo de Góes (IMPG), Universidade Federal do Rio de Janeiro (UFRJ), Rio de Janeiro 21941901, Brazil; thaispdmello@ufrj.br (T.P.M.); simonesantiagorj@yahoo.com.br (S.S.O.); giovanini@micro.ufrj.br (L.G.); mbranquinha@micro.ufrj.br (M.H.B.); 2 Institute for Hygiene and Medical Microbiology, Medical University of Innsbruck, Schöpfstrasse 41, 6020 Innsbruck, Austria; michaela.lackner@i-med.ac.at; 3 Rede Micologia RJ—Fundação de Amparo à Pesquisa do Estado do Rio de Janeiro (FAPERJ), Rio de Janeiro 21941901, Brazil

**Keywords:** siderophores, pyoverdine, phenazines, cystic fibrosis, virulence, microbial interactions

## Abstract

*Pseudomonas aeruginosa* and *Scedosporium/Lomentospora* often coexist in the lungs of cystic fibrosis patients, where their interaction can affect disease outcomes. Our group has recently demonstrated that *P. aeruginosa* suppresses the growth of *Scedosporium/Lomentospora* species partly through mechanisms involving iron sequestration. In this study, we have investigated how molecules secreted by *P. aeruginosa* under high (36 µM) and low (3.6 µM) iron conditions affect the planktonic growth and biofilm formation by *S. apiospermum*, *S. minutisporum*, *S. aurantiacum* and *L. prolificans*. Although *P. aeruginosa* exhibited enhanced proliferation under high-iron conditions, spectrophotometric analyses revealed a marked increase in phenazine and pyoverdine production under low-iron conditions, with siderophore activity confirmed by Chrome Azurol S assays. Supporting these findings, supernatants from *P. aeruginosa* cells grown under iron limitation markedly inhibited fungal growth (≈30%) and biofilm formation (≈70%), whereas those from high-iron cultures were less effective. Notably, low-iron bacterial-free supernatants exhibited pronounced cytotoxic effects on mammalian cells, reducing metabolic activity by an average of 20% in A549 lung epithelial cells and 40% in THP-1 macrophages, and significantly compromising survival in the *Tenebrio molitor* infection model, resulting in 100% larval mortality within 7 days. Collectively, these results indicate that the antifungal activity of *P. aeruginosa* is closely coupled with increased host toxicity. Moreover, the results demonstrate that environmental iron availability plays a critical role in modulating both antifungal activity and toxicity, thereby shaping *P. aeruginosa* interactions with *Scedosporium/Lomentospora* species. Such iron-dependent dynamics may influence the progression and severity of respiratory co-infections, with important implications for patient management and therapeutic interventions.

## 1. Introduction

Cystic fibrosis (CF) is a severe genetic disorder that affects multiple organs, but it is most notably characterized by the production of abnormally viscous mucus in the respiratory tract, creating a favorable niche for persistent microbial colonization and recurrent infections [1]. Among bacterial pathogens, *Pseudomonas aeruginosa* is one of the most prevalent and clinically significant, frequently persisting in the airways of CF patients throughout their lifetime [1,2]. Regarding filamentous fungi, species of the genera *Scedosporium* and *Lomentospora* represent the second most frequently recovered group from CF airways, often co-isolated with *P. aeruginosa* [3,4,5]. Colonization by these fungi typically begins with conidial inhalation, followed by hyphal growth and the establishment of biofilm-like structures that can persist for extended periods. These persistent fungal communities may progress to chronic bronchitis, allergic bronchopulmonary mycosis or even systemic dissemination in patients with severe underlying risk factors [6,7]. Management of such infections is particularly challenging because *Scedosporium/Lomentospora* species display intrinsic multidrug resistance, rendering most clinically available antifungal agents ineffective [3]. Moreover, in vitro studies simulating CF-like conditions have revealed an enhanced resistance of these fungi to azoles, including voriconazole, the current drug of choice, thereby further complicating therapeutic management [8].

Iron is a vital micronutrient required for a wide range of cellular processes, including respiration, DNA synthesis and metabolism. However, iron availability within the human host is tightly restricted, as this metal is typically sequestered by hemoproteins or tightly bound to host chelators such as transferrin and lactoferrin [9]. In CF, this finely tuned balance is frequently disrupted. The sputum of CF patients often contains markedly elevated levels of free and bioavailable iron compared to that of healthy individuals, a consequence of chronic airway inflammation, recurrent tissue damage and pulmonary microhemorrhages [10,11]. These abnormal iron fluctuations profoundly influence the physiology of airway-associated microorganisms, shaping their metabolic activity, virulence traits and competitive interactions within the polymicrobial community of the CF lung [12]. Throughout their evolution, microorganisms have developed highly specialized strategies to acquire iron and outcompete other members of their environment, with the secretion of siderophores being among the most prominent [9,13]. Siderophores are low-molecular-weight secondary metabolites that chelate ferric iron with high affinity, allowing microbial cells to overcome host-mediated nutritional immunity and thrive in iron-limited niches [14]. In *P. aeruginosa*, the major siderophores pyoverdine and pyochelin play central roles not only in nutrient acquisition but also in shaping competitive dynamics within polymicrobial communities [15]. Our group has previously shown that, under CF-mimicking iron-restricted conditions, *P. aeruginosa* suppresses the growth of *Scedosporium* and *Lomentospora* species primarily by depriving them of iron through siderophore-mediated sequestration [16]. Similar iron-dependent antagonistic mechanisms have also been reported in *P. aeruginosa* interactions with other filamentous fungi, including *Aspergillus fumigatus* and *Rhizopus microsporus* [17,18]. Despite these insights, it remains unclear how elevated iron concentrations, such as those frequently observed in CF airways, modulate bacterial proliferation, host-associated toxicity and the competitive dynamics between *P. aeruginosa* and *Scedosporium/Lomentospora* species. Moreover, it is not yet understood whether increased iron availability attenuates, enhances or qualitatively reshapes the antagonistic activity of bacterial siderophores within this polymicrobial niche.

In this study, we have explored the role of iron availability on the interaction between *P. aeruginosa* and clinically significant filamentous fungi associated with CF. We specifically evaluated the effects of *P. aeruginosa*-secreted molecules on *Scedosporium apiospermum*, *S. minutisporum*, *S. aurantiacum* and *Lomentospora prolificans* growth under CF-mimicking conditions with low (3.6 µM) and high (36 µM) iron concentrations. Furthermore, we assessed the cytotoxic of the most deleterious *P. aeruginosa* supernatant on mammalian cell lines and its pathogenicity in the *Tenebrio molitor* larvae infection model. By integrating in vitro and in vivo approaches, our findings reveal how iron fluctuations shape bacterial–fungal interactions in the CF lung environment, offering new perspectives on disease progression and potential therapeutic interventions.

## 2. Materials and Methods

### 2.1. Microorganisms and Culture Conditions

For all experiments conducted in this study, we employed the following clinical isolates obtained from CF patients: *S. apiospermum* strain 12-07, *S. minutisporum* strain 10-28, *S. aurantiacum* strain 11-15 and *L. prolificans* strain 12-19 [16]. Fungal cultures were maintained in modified liquid Sabouraud medium (2% glucose, 1% peptone and 0.5% yeast extract) and preserved by biweekly subculturing, under continuous agitation at 100 rpm and incubation at room temperature [19]. For *P. aeruginosa*, three strains were employed: the reference strain ATCC 27853 and two clinical isolates (8737A and 8737B) obtained from a single CF patient and kindly provided by Dr. Elizabeth de Andrade Marques (Hospital Pedro Ernesto, State University of Rio de Janeiro, Brazil). Bacterial cells were first cultured on Cetrimide agar (Sigma-Aldrich, St. Louis, MO, USA) for 24 h at 37 °C and subsequently transferred to brain heart infusion (BHI) broth (BD Biosciences, Silver Spring, MD, USA), in which cells were incubated for an additional 24 h at 37 °C under constant agitation (130 rpm). Final cultures were adjusted to yield a working suspension of approximately 10^8^ colony-forming unit (CFU)/mL [20].

### 2.2. CF-Mimicking Medium

Synthetic cystic fibrosis sputum medium (SCFM) was prepared as previously described [21], containing 3.6 µM FeSO_4_ (Sigma-Aldrich, St. Louis, MO, USA). To generate the high-iron condition (36 µM), an additional 32.4 µM FeSO_4_ was supplemented to the standard SCFM formulation. Both media were sterilized by filtration through 0.22-μm pore-size membranes (Millipore, São Paulo, SP, Brazil), and the pH was adjusted to 7.0 prior to use.

### 2.3. Preparation of P. aeruginosa Supernatants

To obtain *P. aeruginosa* culture supernatants, bacterial suspensions prepared as described above were inoculated into SCFM containing either 3.6 µM or 36 µM FeSO_4_ at an initial density of approximately 10^6^ CFU/mL. Cultures were incubated at 37 °C with constant agitation (120 rpm) for 72 h. At the end of the incubation period, aliquots were collected from each condition, and bacterial growth was assessed by measuring optical density at 600 nm (OD_600_) using a microplate reader (Multiskan SkyHigh; Thermo Scientific, Waltham, MA, USA). Each culture was then centrifuged (30 min, 4000 rpm, 4 °C), and the resulting cell-free supernatants (designated Sup-3.6 and Sup-36, respectively) were sterilized by filtration through 0.22-μm pore-size membranes (Millipore, São Paulo, SP, Brazil) to ensure the complete removal of bacterial cells.

### 2.4. Quantification of Phenazines, Pyoverdine and Siderophores Activity

Cell-free supernatants were initially scanned using a Multiskan SkyHigh spectrophotometer (Thermo Fisher Scientific, Waltham, MA, USA) across the wavelength range of 340–500 nm to assess the overall spectral profile of secreted metabolites. Specific quantifications of phenazines and pyoverdines were then performed by measuring absorbance at 360 nm and 405 nm, respectively, with the same instrument. To account for variations in bacterial biomass, absorbance values were normalized to growth by calculating the ratios OD_360_/OD_600_ for phenazines and OD_405_/OD_600_ for pyoverdines, as previously described [22]. The siderophore activity was measured using the Chrome Azurol S dye (Sigma-Aldrich, St. Louis, MO, USA) assay, as previously described by Schwyn and Neilands [23].

### 2.5. Preparation of Conidial Cells

Fungal isolates were cultured on potato dextrose agar (PDA; Neogen, Lexington, KY, USA) plates and incubated at room temperature for 7 days to allow conidiation. Conidial suspensions were prepared by gently scraping the colony surface with 5 mL of sterile phosphate-buffered saline (PBS; 150 mM NaCl, 20 mM sodium phosphate, pH 7.2). The resulting suspensions were filtered through a 40-μm nylon mesh (BD Biosciences, Silver Spring, MD, USA) to remove hyphal fragments, followed by three consecutive washes with PBS and centrifugation at 10,000× *g* for 10 min. The final conidial pellets were resuspended in PBS, and conidial concentrations were determined using a Neubauer hemocytometer under light microscopy [16].

### 2.6. Effects of P. aeruginosa Supernatants on Fungal Growth

Conidia (10^4^ per well) were incubated for 24 h at 37 °C in SCFM supplemented with either 3.6 or 36 µM FeSO_4_ (controls) as well as in cell-free bacterial supernatants (designated as Sup-3.6 and Sup-36). Fungal growth was subsequently assessed by measuring absorbance at 600 nm using a Multiskan SkyHigh spectrophotometer (Thermo Fisher Scientific, Waltham, MA, USA) [24].

### 2.7. Effects of P. aeruginosa Supernatants on Fungal Biofilm Formation

Conidia (10^4^ per well) were incubated for 72 h at 37 °C in SCFM supplemented with either 3.6 or 36 µM FeSO_4_ (controls) as well as in cell-free bacterial supernatants (Sup-3.6 and Sup-36). After biofilm formation, biomass, extracellular matrix (ECM) and metabolic activity were quantified. Biofilm biomass was quantified in methanol-fixed samples using classical crystal violet staining (0.4%), with absorbance measured at 590 nm [25]. ECM content was assessed in living, non-fixed biofilms via passive safranin incorporation (0.1%), with absorbance read at 530 nm [26]. Metabolic activity, as a proxy for cell viability, was assessed in living, non-fixed biofilms using the 2,3-bis(2-methoxy-4-nitro-5-sulfophenyl)-2H-tetrazolium-5-carboxanilide (XTT) reduction assay. Biofilms were incubated with XTT (1 mg/mL) supplemented with menadione (0.4 mM; Sigma-Aldrich, St. Louis, MO, USA), and the resulting formazan product was quantified by measuring absorbance at 492 nm [27].

### 2.8. Supernatant Cytotoxicity on Mammalian Cells

A549 (ATCC CCL-185, human alveolar basal epithelial adenocarcinoma) and THP-1 (ATCC TIB-202, human monocytic leukemia) cells were cultured in 75-cm^2^ sterile flasks containing RPMI-1640 (Sigma-Aldrich, St. Louis, MO, USA) supplemented with 10% heat-inactivated fetal bovine serum (FBS; Cultilab, São Paulo, Brazil) at 37 °C in a humidified atmosphere with 5% CO_2_ for 24 h. For these experiments, 10^5^ mammalian cells were seeded per well in 96-well plates containing RPMI-1640 with 10% FBS. After adhesion, wells were washed with RPMI-1640 to remove non-adherent mammalian cells, and 200 µL of either *P. aeruginosa* supernatants (Sup-3.6 and Sup-36), SCFM (control) or RPMI-1640 medium with 10% FBS (control) were added. Following 24 h incubation under the same conditions, mammalian cell viability was assessed using the 3-(4,5-dimethylthiazol-2-yl)-2,5-diphenyl tetrazolium bromide (MTT; Sigma-Aldrich, St. Louis, MO, USA) assay. Plates were incubated in the dark for 3 h at 37 °C to allow metabolically active cells to reduce MTT to purple formazan crystals. Formazan was subsequently solubilized in 100 µL dimethyl sulfoxide (DMSO; Sigma-Aldrich, St. Louis, MO, USA), and absorbance was measured at 450 nm using a ThermoMax microplate reader (Molecular Devices, San Jose, CA, USA) [16].

### 2.9. Supernatant Cytotoxicity in In Vivo Model

*Tenebrio molitor* larvae were selected based on uniform weight (100–150 mg), and individuals exhibiting darkened or damaged cuticles were excluded. Prior to inoculation, larvae were chilled on ice for approximately 5 min to reduce mobility and facilitate precise injection. Using insulin syringes (BD Ultra-Fine 31G, 6 mm, Franklin Lakes, NJ, USA), 10 µL of *P. aeruginosa* supernatants (Sup-3.6 and Sup-36) were injected into the hemocoel via the ventral surface through the fifth sternite. Following inoculation, larvae were maintained in the dark at 37 °C for 7 days, with food provided ad libitum and daily monitoring of survival. Mortality was determined by the absence of response to gentle tactile stimulation, typically accompanied by extensive melanization of the cuticle, serving as an additional indicator of infection progression [28].

### 2.10. Statistical Analysis

All experiments were performed at least three times, using biological triplicates. Results are expressed as mean ± standard deviation. Statistical analyses were performed using ANOVA followed by Sidak’s, Tukey’s or Dunnett’s multiple comparisons test and the survival curves were generated using the Kaplan–Meier method. All analyses were performed with GraphPad Prism 9 software. *p*-values ≤ 0.05 were considered statistically significant.

## 3. Results

### 3.1. Iron Availability Modulates P. aeruginosa Growth and Siderophore Production

**Figure 1:**
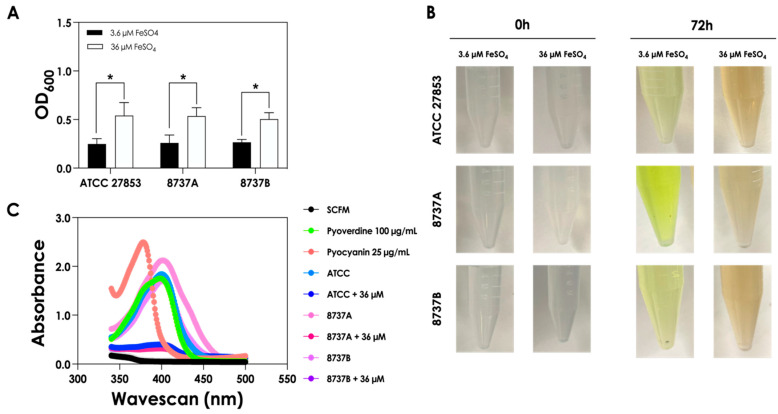
Growth and metabolite profile of *P. aeruginosa* under varying iron concentrations. (**A**) Optical density at 600 nm (OD_600_) of *P. aeruginosa* cultures grown for 72 h in SCFM containing 3.6 µM or 36 µM FeSO_4_ at 37 °C with constant agitation (120 rpm). Asterisks (*) indicate statistically significant differences between high- and low-iron conditions (*p* < 0.0001; two-way ANOVA with Sidak’s multiple comparisons test). (**B**) Representative images showing the color of culture supernatants after 72 h, illustrating the green fluorescence at low-iron and brownish hue at high-iron. (**C**) Absorbance spectra (340–500 nm) of cell-free supernatants collected after 72 h, highlighting the characteristic pyoverdine peak under low-iron conditions and its reduction under high-iron conditions. In this figure, the dark purple curve (representing the 8737B + 36 µM system) is completely overlapped by the other curves and is therefore not distinguishable. Note: ATCC, 8737A and 8737B refer to *P. aeruginosa* strains.

We have previously demonstrated that, under conditions mimicking the CF lung, *P. aeruginosa* inhibits the growth of *S. apiospermum*, *S. minutisporum*, *S. aurantiacum* and *L. prolificans* primarily through the production of pyoverdines [16]. In the CF lung, however, iron availability can fluctuate—for instance, due to the microhemorrhages—potentially altering fungal-bacterial interactions. Building on our previous work, we herein analyzed the profiles of *P. aeruginosa* supernatants obtained under low (3.6 µM; Sup-3.6) and high (36 µM; Sup-36) iron concentrations. We first assessed the growth of three *P. aeruginosa* strains under these conditions (Figure 1). All bacterial strains exhibited significantly higher growth in SCFM supplemented with 36 µM FeSO_4_ compared to 3.6 µM, indicating that elevated iron concentration promotes bacterial proliferation (Figure 1A). After 72 h, the Sup-3.6 displayed a characteristic fluorescent green coloration, whereas the Sup-36 appeared brownish (Figure 1B). Absorbance profiling of cell-free bacterial supernatants revealed that those obtained under low-iron conditions closely resembled purified pyoverdine, while supernatants from high-iron cultures lost this signature (Figure 1C), suggesting that increased iron availability modulates siderophore production and overall metabolite composition.

### 3.2. Iron-Dependent Regulation of Phenazine, Pyoverdine and Siderophore Production in P. aeruginosa

**Figure 2:**
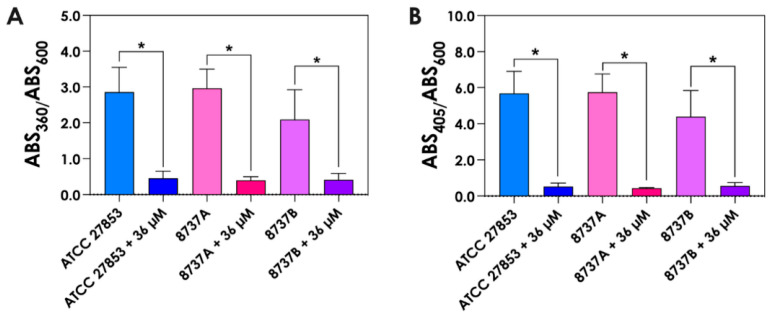
Quantification of phenazine and pyoverdine production by *P. aeruginosa* under different iron conditions. 72 h-culture supernatants from *P. aeruginosa* grown in SCFM containing either 3.6 µM or 36 µM FeSO_4_ were collected, filtered, and analyzed for absorbance at 360 nm (**A**) for phenazines and 405 nm (**B**) for pyoverdines. Values were normalized to bacterial growth (OD_600_) under each condition. Asterisks (*) indicate statistically significant differences between low- and high-iron conditions (*p* < 0.05; one-way ANOVA with Tukey’s multiple comparisons test). ATCC, 8737A and 8737B refers to *P. aeruginosa* strains.

To validate the shift in the molecular profile of *P. aeruginosa* supernatants (Sup-3.6 and Sup-36) observed in Figure 1B under elevated iron conditions, we quantified the levels of phenazines (Figure 2A) and pyoverdines (Figure 2B). Both classes of metabolites were significantly reduced in supernatants from cultures grown with 36 µM FeSO_4_ compared to those grown under low-iron conditions (3.6 µM FeSO_4_), confirming that increased iron availability suppresses siderophore and phenazine production.

**Figure 3:**
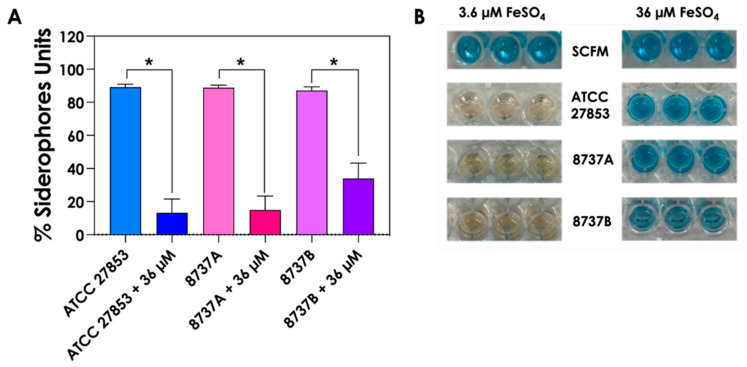
Siderophore activity of *P. aeruginosa* cultured under different iron concentrations. (**A**) Twenty-four-hour culture supernatants from *P. aeruginosa* grown in SCFM containing either 3.6 µM or 36 µM FeSO_4_ were collected, filtered, and siderophore activity was measured using the chrome azurol S (CAS) assay. Values are shown as mean ± SD. Asterisks (*) indicate statistically significant differences between low- and high-iron conditions (*p* < 0.05; one-way ANOVA with Tukey’s multiple comparisons test). (**B**) Representative images of the CAS assay after 1 h, illustrating higher siderophore activity in supernatants from low-iron cultures compared to high-iron cultures. ATCC 27853, 8737A and 8737B refers to *P. aeruginosa* strains.

Following the observed differences in pyoverdine concentrations between supernatants obtained under low- (3.6 µM) and high (36 µM)-iron conditions, we next assessed the total siderophore activity of *P. aeruginosa* cultures. Consistent with the earlier measurements, overall siderophore activity was markedly reduced in supernatants from bacteria grown in 36 µM FeSO_4_ compared to those cultured under low-iron conditions (Figure 3A,B). These results further support the conclusion that elevated iron availability suppresses siderophore production, which may have significant implications for nutrient competition and microbial interactions in iron-rich microenvironments.

### 3.3. Iron-Dependent Inhibition of Fungal Growth and Biofilm Formation by P. aeruginosa

**Figure 4:**
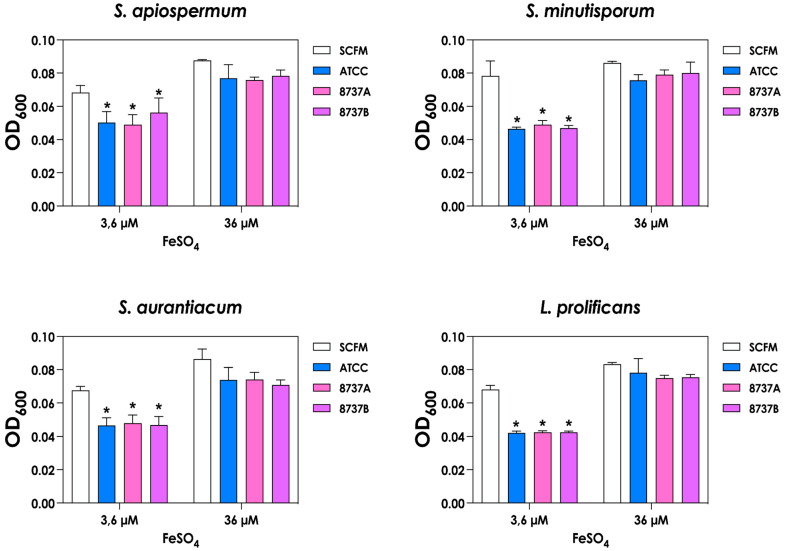
Effects of *P. aeruginosa* supernatants obtained under different iron conditions on the growth of *Scedosporium*/*Lomentospora* species. Conidia (1 × 10^4^) were incubated for 24 h at 37 °C in SCFM or in *P. aeruginosa* supernatants obtained from cultures grown with either 3.6 µM or 36 µM FeSO_4_. Fungal growth was assessed by measuring optical density at 600 nm (OD_600_). Asterisks (*) denote statistically significant differences between fungal growth in the presence of supernatants and respective SCFM controls (*p* < 0.05; two-way ANOVA with Sidak’s multiple comparisons test). ATCC, 8737A and 8737B refers to *P. aeruginosa* strains.

Previous studies have shown that phenazines and siderophores produced by *P. aeruginosa* play a key role in inhibiting fungal growth during co-culture [29,30,31,32,33,34,35,36,37,38]. To determine whether this mechanism extends to *S. apiospermum*, *S. minutisporum*, *S. aurantiacum* and *L. prolificans*, we evaluated fungal growth following exposure to *P. aeruginosa* supernatants obtained from cultures grown in SCFM under low- (3.6 µM) and high (36 µM)-iron conditions. In all cases, fungal growth was significantly reduced in the presence of Sup-3.6 compared to Sup-36 bacterial secretions (Figure 4), consistent with the higher abundance of inhibitory molecules under iron-limited conditions. These results reinforce the central role of iron-dependent metabolites, such as pyoverdines and phenazines, in mediating bacterial–fungal antagonism in environments resembling the CF lung.

**Figure 5:**
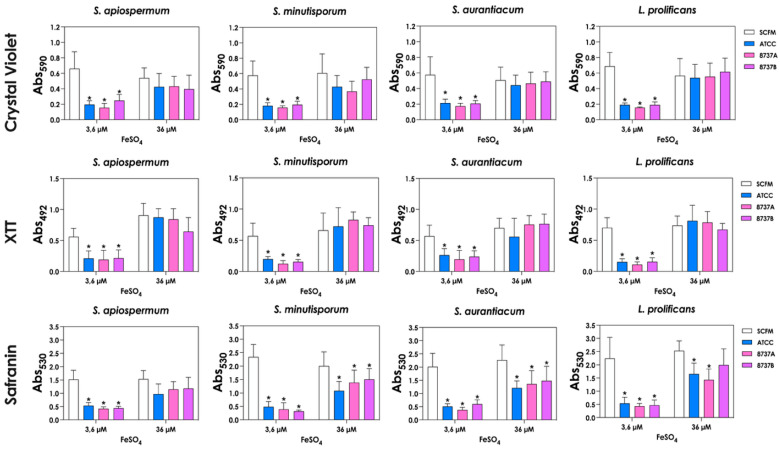
Impact of *P. aeruginosa* supernatants obtained under different iron conditions on biofilm formation by *Scedosporium*/*Lomentospora* species. Conidia (1 × 10^4^) were incubated for 72 h at 37 °C in *P. aeruginosa* supernatants (Sup-3.6 and Sup-36). Biofilms were subsequently analyzed using crystal violet staining for biomass quantification, XTT reduction for metabolic activity, and safranin staining for extracellular matrix assessment. Asterisks (*) denote statistically significant differences between biofilms formed in SCFM controls and in supernatants obtained from cultures grown in SCFM with either 3.6 µM or 36 µM FeSO_4_ (*p* < 0.05; two-way ANOVA with Dunnett’s multiple comparisons test). ATCC 27853, 8737A and 8737B refer to *P. aeruginosa* strains.

Finally, we assessed biofilm formation by *S. apiospermum*, *S. minutisporum*, *S. aurantiacum* and *L. prolificans* in the presence of *P. aeruginosa* supernatants obtained under different iron conditions. Three classical biofilm parameters (biomass, metabolic activity and ECM content) were evaluated. Biofilms formed in the presence of supernatants from low-iron cultures exhibited reductions in all three parameters compared to controls, indicating a strong inhibitory effect on biofilm development (Figure 5). In contrast, supernatants from high-iron cultures had a more selective impact, significantly reducing only ECM production in *S. minutisporum*, *S. aurantiacum* and *L. prolificans*, while biomass and metabolic activity remained largely unaffected (Figure 5). These findings suggest that the iron-dependent production of bacterial metabolites modulates not only fungal growth but also the structural and functional characteristics of fungal biofilms.

### 3.4. Cytotoxicity and Pathogenic Effects of P. aeruginosa Supernatants Under Iron-Limited Conditions

**Figure 6:**
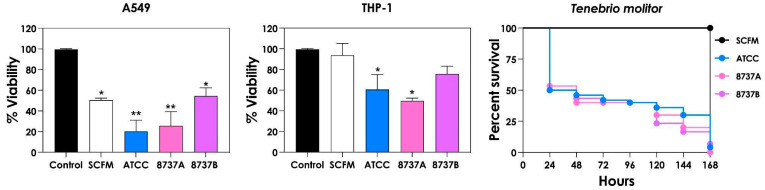
Effect of *P. aeruginosa* supernatants on mammalian cell lines and *Tenebrio molitor*. A549 and THP-1 cells were incubated for 24 h at 37 °C with 5% CO_2_ in the presence of *P. aeruginosa* supernatants. Cellular metabolic activity was assessed by MTT reduction assay. Asterisks indicate significant differences between cells exposed to RPMI-1640 with 10% FBS (control) compared to SCFM and those treated with bacterial supernatants (* *p* < 0.05; ** *p* < 0.005; two-way ANOVA, Dunnett’s multiple comparisons test). *T. molitor* larvae (n = 10 per group) were inoculated with *P. aeruginosa* supernatants, and survival was monitored daily for 7 days. Survival rates were constructed using the Kaplan–Meier curve.

Once the *P. aeruginosa* supernatant obtained from SCFM containing 3.6 µM FeSO_4_ exhibited the strongest inhibitory effect on fungal cells, we next assessed its impact on mammalian cells (A549 and THP-1) as well as in the in vivo *T. molitor* larvae model (Figure 6). After 24 h exposure, only the conditioned supernatants from the ATCC 27853 and 8737A strains caused significant cytotoxicity in both THP-1 macrophages and A549 lung epithelial cells compared to controls cultured in either RPMI or SCFM media (Figure 6). In contrast, in the in vivo model, supernatants from all three strains induced complete mortality of larvae within 7 days (Figure 6), highlighting the potent pathogenic potential of bacterial secreted factors under iron-limited conditions.

## 4. Discussion

Iron availability in the cystic fibrosis (CF) lung is highly dynamic and critically shapes microbial interactions. In a previous study, we showed that under CF-mimicking low-iron conditions, *P. aeruginosa* suppresses the growth of *Scedosporium*/*Lomentospora* species mainly through the secretion of the siderophores pyoverdine and pyochelin, which limit fungal access to iron [16]. Building on these findings, the present work demonstrates that iron availability not only modulates siderophore production but broadly alters the repertoire of antifungal metabolites secreted by *P. aeruginosa*, thereby influencing fungal growth, biofilm formation and host toxicity.

As expected, bacterial growth was enhanced under iron-replete conditions (36 µM FeSO_4_), whereas low iron (3.6 µM FeSO_4_) promoted the secretion of iron-regulated secondary metabolites. Supernatants from low-iron cultures exhibited the characteristic spectral signature of pyoverdine, while this signal was absent under high-iron conditions, consistent with the strict iron-dependent regulation of siderophore biosynthesis. Notably, low iron also led to increased absorbance at 360 nm, indicative of phenazine accumulation. Unlike previous observations in specific *P. aeruginosa* strains where iron repletion shifts antifungal activity from siderophores to phenazines [22], our data indicate that iron limitation simultaneously stimulates both siderophore and phenazine production, suggesting a coordinated rather than substitutive antifungal strategy. This enhanced metabolic output under low-iron conditions translated into markedly stronger antifungal effects. Supernatants rich in siderophores and phenazines were significantly more inhibitory to the growth and biofilm formation of *S. apiospermum*, *S. minutisporum*, *S. aurantiacum* and *L. prolificans* than those produced under iron-replete conditions. While siderophore-mediated iron sequestration plays a central role in this antagonism [16], previous co-culture studies showed that inhibition of siderophore biosynthesis only partially restores fungal growth, indicating that additional factors contribute to fungal suppression. In this context, phenazines likely represent key complementary effectors. Adding further complexity, contrasting evidence suggests that siderophores may not always be detrimental to fungi. In particular, Le Govic et al. [39] reported that *S. apiospermum* could exploit pyoverdine as a xenosiderophore, turning a typically inhibitory molecule into a nutritional advantage. Reconciling these apparently contradictory findings remains an open question, and our group is currently addressing this issue in collaboration with Prof. Jean-Philippe Bouchara (Université d’Angers, France). Taken together, these observations support the notion that siderophores alone cannot fully account for the inhibitory activity of *P. aeruginosa* against *Scedosporium* and *Lomentospora*. Additional metabolites, particularly phenazines, likely contribute to the antifungal effects. Indeed, phenazines are considered among the most important *P. aeruginosa* virulence factors, mediating toxicity against both prokaryotic and eukaryotic organisms through their redox activity and capacity to induce oxidative stress [35]. The enrichment of phenazines in supernatants derived from low-iron environments underscores their potential role as key effectors in the suppression of fungal growth and biofilm formation under cystic fibrosis-like conditions.

Phenazines are redox-active metabolites with well-documented antifungal activity mediated by oxidative stress induction, membrane damage and disruption of iron homeostasis. Consistent with earlier reports, phenazines impair fungal germination and growth in several pathogenic fungi, including *Scedosporium* species [35,40,41,42,43]. However, their effects are context dependent, as subinhibitory concentrations may paradoxically promote fungal growth under iron limitation by increasing iron bioavailability. These dual roles underscore the ecological complexity of bacterial-fungal interactions and highlight iron availability as a critical determinant of phenazine activity.

Importantly, the potent antifungal activity of low-iron *P. aeruginosa* supernatants was accompanied by pronounced cytotoxicity toward mammalian cell lines and invertebrate hosts. This finding is clinically relevant, as phenazines and siderophores are abundant in CF sputum and their concentrations correlate with disease severity [33]. Phenazines such as pyocyanin and 1-hydroxyphenazine are known to disrupt cellular respiration, induce excessive reactive oxygen species and impair key host defense mechanisms, while siderophores can further exacerbate toxicity by disturbing iron homeostasis [44,45,46,47,48,49,50,51,52,53,54]. Thus, the same metabolites that suppress fungal competitors may simultaneously contribute to host tissue damage and disease progression.

In summary, our results demonstrate that iron availability is a major determinant of the metabolic and antagonistic behavior of *P. aeruginosa* under CF-like conditions. Low-iron environments promote the coordinated production of siderophores and phenazines, enhancing antifungal activity against *Scedosporium*/*Lomentospora* species, but at the cost of increased host toxicity. These findings emphasize the dual and context-dependent roles of *P. aeruginosa* secondary metabolites in polymicrobial CF infections and highlight iron as a key ecological driver shaping microbial interactions and host outcomes. It is important to note, however, that the absence of fractionation and metabolomics-based analyses in the present study precludes the resolution of individual molecular species or structural variants potentially involved in iron chelation and antifungal activity. The approaches employed here were designed to capture global biological effects rather than to define the precise composition or relative contribution of specific metabolites. Accordingly, future studies integrating chemical fractionation and metabolomic strategies will be essential to comprehensively characterize the metabolites involved and to refine the mechanistic basis of these interactions. A deeper understanding of these iron-dependent dynamics may ultimately inform therapeutic strategies aimed at limiting both fungal colonization and bacteria-driven tissue damage in CF patients.

## Data Availability

The original contributions presented in this study are included in the article. Further inquiries can be directed to the corresponding author.
